# Physicochemical characterization of porcine respiratory aerosol and considerations for future aerovirology

**DOI:** 10.1093/pnasnexus/pgad087

**Published:** 2023-03-22

**Authors:** Robert Groth, Sadegh Niazi, Kirsten Spann, Graham R Johnson, Zoran Ristovski

**Affiliations:** School of Earth and Atmospheric Sciences, International Laboratory for Air Quality and Health, Faculty of Science, Queensland University of Technology, Brisbane, QLD 4000, Australia; School of Earth and Atmospheric Sciences, International Laboratory for Air Quality and Health, Faculty of Science, Queensland University of Technology, Brisbane, QLD 4000, Australia; School of Biomedical Sciences, Centre for Immunology and Infection Control, Faculty of Health, Queensland University of Technology, Brisbane, QLD 4000, Australia; School of Earth and Atmospheric Sciences, International Laboratory for Air Quality and Health, Faculty of Science, Queensland University of Technology, Brisbane, QLD 4000, Australia; School of Earth and Atmospheric Sciences, International Laboratory for Air Quality and Health, Faculty of Science, Queensland University of Technology, Brisbane, QLD 4000, Australia

**Keywords:** respiratory aerosol, droplet physicochemistry, virus viability

## Abstract

Understanding the mechanisms which inactivate airborne viruses is a current challenge. The composition of human respiratory aerosol is poorly understood and needs to be adequately investigated for use in aerovirology studies. Here, the physicochemical properties of porcine respiratory fluid (PRF) from the trachea and lungs were investigated both in bulk solutions and in aerosols. The mass ratio of Na:K in PRF compared with cell culture media (Dulbecco's Modified Eagle Medium, DMEM), which is frequently used in aerovirology studies, was significantly lower (∼2:1 vs ∼16:1). PRF contained significantly more potassium and protein than DMEM. PRF aerosols of all samples were similarly hygroscopic to human respiratory aerosol. PRF particles could nucleate with spatially separated crystals, indicating that the protein matrix was sufficiently viscous to prevent the complete coalescence of aqueous salts prior to efflorescence. The effects of these differences in compositions on the viability of viruses are currently not well understood. The virus suspensions in aerovirology studies need to be reconsidered to adequately reflect a real-world expiration scenario.

**Significance Statement**

Determining the mechanisms of aerosol phase virus inactivation is a current major research objective in aerovirology. The knowledge of these mechanisms, however, can only be extended to reality along with the comprehensive understanding of the physical and chemical properties of the respiratory aerosols in which these viruses are embedded. This work provides unique and detailed physicochemical characterization of respiratory aerosol in comparison with a commonly used respiratory aerosol surrogate. In all measured metrics, the authentic respiratory fluid was statistically different to the surrogate. This work provides useful framework on which to base respiratory fluid surrogates in further aerovirology research.

## Introduction

The global burden of viruses on human health is of great concern, exacerbated by the severe acute respiratory syndrome coronavirus 2 (SARS-CoV-2) pandemic (1, 2). Along with SARS-CoV-2, other viruses such as influenza A virus, human rhinovirus, and measles virus can be transmitted between individuals through respiratory aerosols or ballistic droplets (3, 4). Along with airborne routes, direct contact (interpersonal contact) and indirect contact (fomite) also contribute to the transmission of these pathogens. The exact proportion of the airborne and contact routes to the net transport of respiratory viruses is not well understood, but it has been reported that protective measures such as face masks have been effective measures of limiting the spread of SARS-CoV-2 to varying degrees of efficacy (5–7). The ability of an airborne virus to survive and transmit from a host to a susceptible individual depends on the physicochemistry of the respiratory particle in which it is embedded (8), the transmission route, and the intrinsic probability of the virions causing an infection. The expired particle provides the microenvironment for the pathogen, and the chemical properties of the respiratory fluid will affect the size, pH, and salinity of the particle, which can consequently affect the virus infectivity. The virus localization within the particle may then also impact viability depending on whether it is embedded within an organic (protein and lipids) matrix, within an aqueous inorganic (salts) inclusion, at the air–particle interface or at the organic–inorganic interface. The location of the virus upon generation of the particle is likely homogeneously distribution throughout the bulk (9, 10), and as the particle evaporates to reach equilibrium with the atmosphere, the viruses may redistribute throughout the particle in different ways. Upon further dehydration, the viscosity of the protein can increase and then limit the rearrangement of the aqueous inorganics within the particle (11, 12). Initial phase separation between the aqueous organics and inorganics may lead to multiple inorganic inclusions throughout the particle, which, if sufficiently short evaporation timescales, may not coalesce into a single inorganic aqueous inclusion (11). If viruses are positioned at the organic–inorganic interface, then particles with numerous inorganic inclusions will have greater interfacial surface area, meaning there are more sites for the viruses to inhabit. It is clear that there is a relationship between droplet physicochemistry and virus viability, but further studies are required to identify how compositional and morphological factors of the droplet affect virus viability.

Aerovirology studies vary in methodology and research objective but all nebulize the virus in a carrier fluid and then quantify the viable viruses either in the air or after deposition on a substrate. Typically, there is also a conditioning step after nebulization which involves aging of the particles under different climatic conditions. The composition of the carrier fluid will provide the microenvironment of the virus and, thus, is a critical factor when determining the viability and inactivation mechanisms of the viruses. Different aerovirology studies have reported the composition of the nebulization fluid in different ways, which has caused confusion in interpreting the concentration of constituents, namely, the proteins and salts. The mechanism of nebulization may also influence the composition of the aerosols (i.e. the composition of the bulk fluid and the aerosols might differ) through the enrichment of organics. After nebulization, some intermediate storage system is required for the particles to reach equilibrium with some controlled environment. In the case of particle levitation studies which investigate physicochemical properties of a select number of particles, that controlled environment is a sealed chamber in which the humidity and temperature can be altered and maintained (13, 14). For studies which investigate ensembles of particles, a rotating storage chamber is a common choice both to provide a controlled environment and to also minimize particle losses due to gravitational settling (2, 15–18). After storage, the aerosols are then collected either on a solid medium or into a liquid medium (e.g. culture media in impinger). The viability of the viruses in the recovered aerosol can then be determined through viability assays. Each study may use any different combination of these steps, including different growth media, different nebulization methods, different particle sizes, and different methods of virus quantification. Through all of these variations in experimental protocol, it becomes clear that the results between studies may not be easily comparable and more work must be done in understanding the importance of carrier fluid composition on virus viability. Understanding how the physicochemical properties of respiratory particles, including morphology, composition, temperature, relative humidity (RH), and pH, will affect the viability of embedded viruses will allow for interpretation of the results of aerovirology studies. For understanding these properties, porcine respiratory fluid (PRF) is a suitable surrogate for human respiratory fluid (HRF).

The links between the physicochemical properties of the carrier particle which provides the microenvironment for an airborne virus and the eventual fate of the virus are poorly understood. It is not clear how changes in the particle composition will then interact with ambient conditions (RH and temperature) to then affect the salinity or pH to which the virus is exposed. Especially, the contribution of different salt species is unknown. Research is accumulating to understand better the composition and morphology of respiratory aerosols, which will be useful to explain virus viability in aerovirology studies. Recently, work has shown that simulated respiratory fluid (SRF), including simple organic/inorganic solutions and growth media, exhibits semisolid organic phases upon dehydration (9, 12, 13, 19). Numerous studies also confirm the crystallization of SRF (efflorescence) either through hygroscopicity measurements or micrograph analysis (9, 10, 12, 14, 18, 20, 21), and some provided evidence that effloresced salts were less effective at inactivating viruses than concentrated aqueous salts (17). Further, studies have determined that pathogens can exhibit high pH sensitivity and that expired particles may change in pH over their transport (14, 22–24). While similar to human saliva in composition, it is unlikely that growth media are representative of human lung fluid, which is estimated to be upwards of 90% protein by solute volume (12, 20). It is unclear whether the results of these studies translate to droplets composed of real lung fluid.

This research aims to address some of these gaps in the literature. Specifically, we compare the physical and chemical properties of PRF (as an analog for HRF) aerosol with cell growth media aerosol. To ensure that the results of aerovirology studies are consistent with viruses expired by humans in a realistic scenario, it is important to ensure that the pathogen microenvironment is representative of HRF. In this study, we measure and contrast several physical and chemical properties of PRF aerosol and Dulbecco's Modified Eagle Medium (DMEM) aerosol. We determine from this that growth media are not sufficiently representative of respiratory aerosol and viral suspensions should be modified to increase the representativity of the virus microenvironment in aerovirology studies.

## Materials and methods

### Collection and characterization of PRF

PRF was successfully retrieved from *N* = 6 deceased piglets. Fluid was recovered separately from the trachea (T) and the lungs (L). The trachea lining fluid was segregated from the lungs using a cable tie. First, the trachea was washed with ultrapure water (Thermo Fisher Scientific, CN: 10977015). The trachea was then gently massaged manually, and the trachea fluid was recovered using a pipette. The same process was repeated for the lungs to recover the lung fluid. Through the collection process, the recovered PRF was diluted in the collection medium (ultrapure water) to an unknown amount, but from the volume of total recovered fluid and initially introduced fluid, the recovered fluid was estimated to be ∼95% collection water. The exact dilution between samples will vary due to differences in organ geometry and the native volume of PRF in the organs, so it is more appropriate to compare relative concentrations of the constituents. The ion concentration within the recovered PRF was measured using inductively coupled plasma optical emission spectroscopy (ICP-OES, Optima 8300 ICP-OES, PerkinElmer). The ICP-OES was fitted with an ESI SC4DX autosampler and prepFAST 2 sample handling unit for online internal standardization. Calibration standards were used for elemental quantification of the solutions, which were diluted in 2% nitric acid prior to analysis. The wavelength for the Na, K, and Ca concentrations were 589.592, 766.490, and 315.887 nm, respectively. The total protein concentration was measured using a BCA Protein Assay Kit (Thermo Fisher Scientific, CN: 23227) and was used as directed by the manufacturer.

### Surface analysis of growth media aerosols

DMEM (Thermo Fisher Scientific) aerosol was analyzed to compare the physicochemical properties of growth media to PRF. DMEM was nebulized, passed through a silica dryer, and deposited on a Si wafer to produce a thin film to analyze. Crystallographic data of DMEM aerosol were collected using grazing incidence X-ray diffraction (Section S6). The infrared absorbance spectrum of DMEM aerosol was collected using Fourier-transform infrared (FTIR) spectroscopy (Section S7).

### Hygroscopicity measurements

A total of 0.1 mL of PRF was mixed with 4.9 mL of 18.2 MΩcm water and aerosolized using a Collison nebulizer, with filtered and dried compressed air as the carrier gas. The PRF aerosols were passed through a silica diffusion dryer at 0.3 L min^−1^ for 80 cm (residence time ∼80 s). The RH after drying the particles was measured using a RH sensor (HC2-C04, Rotronic AG, Switzerland) to be <5%. The diametric hygroscopic growth factor (GF) of the particles was measured using a humidification tandem differential mobility analyzer (H-TDMA) (25–27) and performed identically to a previous study (12). In short, the dry aerosols were charge neutralized using an ^85^Kr neutralizer and a 100-nm monodisperse aerosol fraction was selected using the first differential mobility analyzer (DMA1). The sheath air flow in DMA1 was dry air at 4.5 L min^−1^. The monodisperse fraction was then passed through a humidity conditioner in either dehydration or hydration cycles. For dehydration cycles, the monodisperse fraction is first prehumidified using a gas exchange cell (FC100-6, Perma Pure LLC, Lakewood, NJ, USA). For hydration cycles, the prehumidification gas exchange cell was bypassed. The aerosol was then passed into the second DMA (DMA2), which had a humid sheath flow of 3.5 L min^−1^. The RH in the DMA2 sheath flow was initially ∼90% and was decreased to <10% during the experiments. After RH conditioning in DMA2, the particle size distribution (PSD) was measured using a condensation particle counter (3776 CPC, TSI, Shoreview, MN, USA). The conditioned PSD data were then inverted using the TDMAinv algorithm (28), and the GF was then calculated as the ratio of the median particle diameter at the conditioned RH (*D*_RH_) to the median particle diameter at RH < 10% (*D*_dry_), as GF = *D*_RH_/*D*_dry_. This process was repeated for each PRF sample as listed in Table 1 and with DMEM. The Na to K (Na:K) mass ratio measured by ICP-OES was used to calculate the relative volume of NaCl and KCl in the dry particle, and the protein volume fraction of dry solute (*φ*_protein_) was estimated using ordinary least-squares fitting with the Zdanovskii–Stokes–Robinson (ZSR) mixing rule (29) using NaCl and KCl data from the AIOMFAC model (https://aiomfac.lab.mcgill.ca) (30, 31). Both porcine gastric mucin (PGM) and bovine serum albumin (BSA) were considered as the proteinaceous component of the particles.

**Table 1.** Modeled and measured solute protein volume fraction (*φ*) for porcine respiratory fluid (PRF) aerosols.

	Modeled			Measured
	ZSR ± 0.01		GF_90_	Bulk
Sample site/ID	*φ*_BSA_	*φ*_PGM_	*φ*_PGM_	*φ*_protein_
L3	0.925	0.929	0.971	0.971
T3	0.919	0.927	0.970	0.977
L5	0.929	0.933	0.973	0.977
T5	0.947	0.951	0.980	0.989
L6	0.939	0.943	0.977	0.986
T6	0.923	0.927	0.969	0.987

### Microscopy

Aerosol samples were collected for transmission electron microscopy (TEM) analysis. Each of the samples (Table 1 and DMEM) was nebulized individually, and particles were passed through the same silica diffusion dryer (RH < 5%) and ^85^Kr neutralizer described in the previous section. High-efficiency particulate air-filtered compressed air was the carrier gas and was measured at 1 L min^−1^ out of the charge neutralizer. The particles were then collected after ∼10 min of deposition onto a TEM grid (continuous carbon coated, 200 mesh copper grid, Ted Pella, Inc.) using a TSI Nanometer Aerosol Sampler 3089 (TSI, Shoreview, MN, USA) at 1 L min^−1^ and −9 kV. TEM was performed using a JEOL 2100 TEM operating at 200 kV of accelerating voltage. Elemental analysis of particles was performed using energy-dispersive X-ray spectroscopy (EDS) using an Oxford Instruments X-Max EDS detector (Oxford Instruments, Oxford, UK), which detects characteristic X-rays emitted from electron excitation during TEM measurement. The aspect ratio (AR) of PRF particles (amount of particle deformation) was used as an indicator for particle phase and was measured using atomic force microscopy (AFM). Particles for AFM analysis (L6, T6) were collected as described above for TEM analysis but were instead collected on Si wafers (Ted Pella, Inc.). The AFM (Bruker Dimension Icon PT AFM, Bruker Co., Billerica, MA, USA) was housed in a vibration isolation chamber in which the RH and temperature were measured to be 35 ± 2% and 26 ± 1°C, respectively, over the course of the experiment, using a silicon nitride AFM probe with a nominal spring constant of 0.4 Nm^−1^ in PeakForce Tapping mode.

## Results

### Compositional analysis

**Fig. 1.**
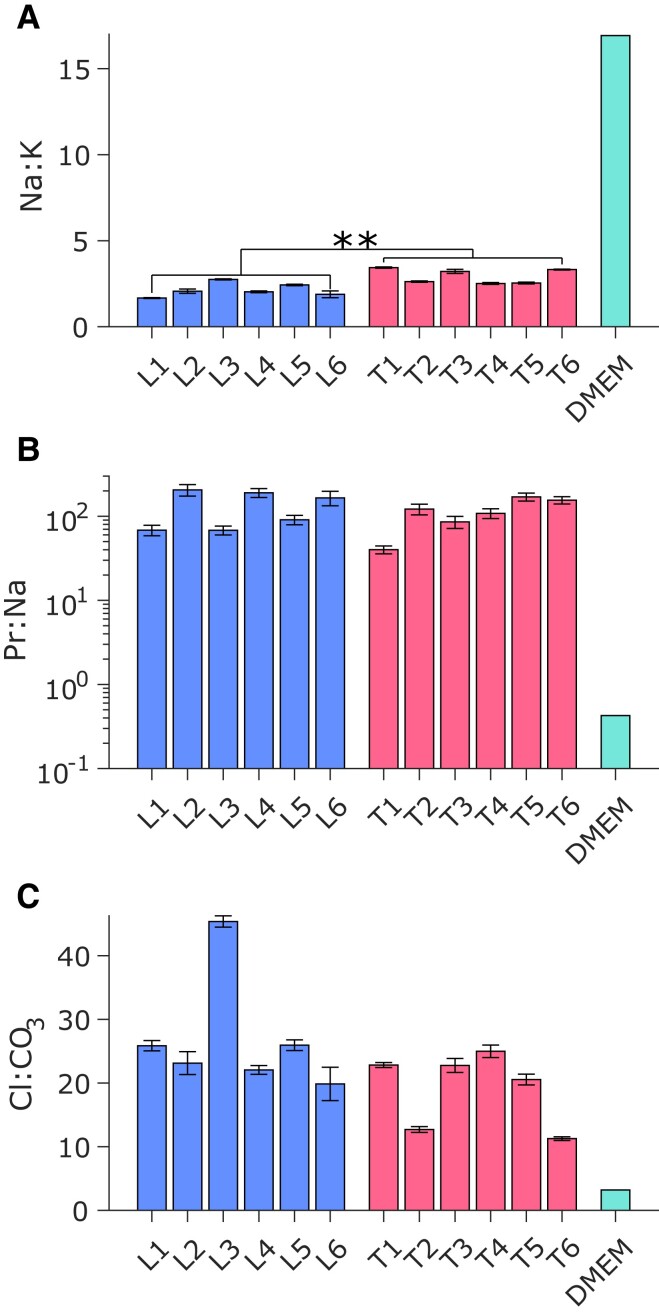
(A) Na:K mass ratio, (B) protein:Na mass ratio (Pr:Na), and (C) estimated Cl:CO_3_ mass ratio of porcine respiratory fluid (PRF) lung (L) and trachea (T) samples and DMEM. For DMEM, the protein mass was considered as the total mass of amino acids, and Cl:CO_3_ for PRF is estimated. Error bars are from the relative standard deviation (RSD) of the inductively coupled plasma optical emission spectroscopy (ICP-OES) data. **P < 0.01.

The elemental composition of PRF from ICP-OES and the total protein concentration using a BCA Protein Assay are tabulated (Table S1). The mass ratio of Na:K ions was found to vary statistically between the lung and trachea samples (*P* < 0.01) (Fig. 1A). The median Na:K for the lung and trachea samples was 2.0 ± 0.2 and 2.9 ± 0.2, respectively. The Na:K ratio for DMEM was higher than that for PRF (∼8-fold and ∼6-fold larger than lung and trachea, respectively) due to lower concentration of potassium salts in DMEM. There was no statistically significant difference in the total protein concentrations between the lung and trachea samples (*P* > 0.9; Table S1). Additionally, the mass ratio of protein to Na (Pr:Na) was calculated for lung and trachea fluid in order to compare with DMEM (Fig. 1B). The median Pr:Na mass ratio in PRF was 110 ± 20 and was considerably larger than that in DMEM (2-log increase). Assuming that each Ca^2+^ ion is in a 1:1 mole ratio with ${\mathrm{CO}}_{3}^{2\text{-}}$ and that all Ca measured by ICP-OES were positively charged, then a lower estimate of the mass ratio of Cl:CO_3_ can also be calculated (14). From these estimates, there was a 1-log higher concentration of Cl than CO_3_ (Fig. 1C) in PRF. This analysis was also conducted for DMEM, and both the carbonate and calcium mass concentration was considerably larger than those in PRF (∼7-fold).

### Hygroscopicity and composition modeling

**Fig. 2.**
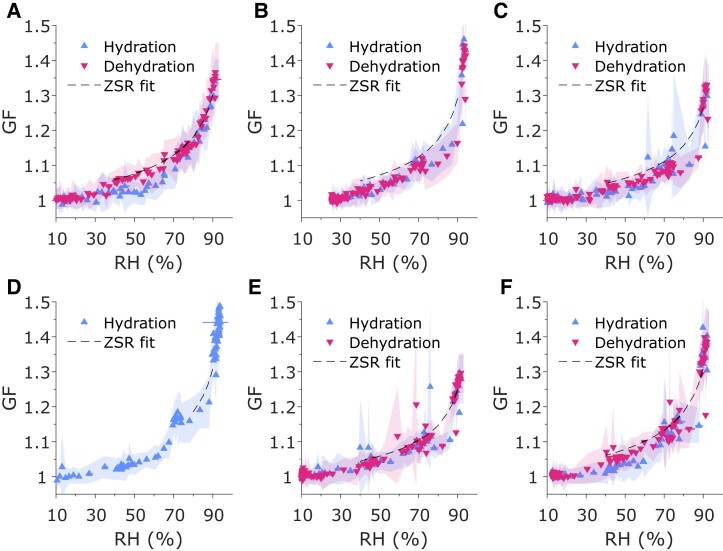
Diametric hygroscopic growth factor (GF) as a function of relative humidity (RH) for porcine respiratory fluid (PRF) aerosols as measured by humidification tandem differential mobility analyzer (H-TDMA) for samples (A) L3, (B) L5, (C) L6, (D) T3, (E) T5, and (F) T6. Shaded regions are standard error of the TDMA inversion.

Hygroscopicity of samples L3, T3, L5, T5, L6, and T6 were measured using H-TDMA (Fig. 2). Efflorescence and deliquescence were observed in sample L3 (Fig. 2A) at ∼30% and ∼70%, respectively, and deliquescence was also observed in sample T3 at ∼70% RH, although experimental noise limits interpretation of the data. The hygroscopic growth was also measured for DMEM (Fig. S2), and it was considerably more hygroscopic (GF_90_ = 1.77) than all PRF samples.

The estimated volume fractions of protein for six H-TDMA data sets were calculated using the ZSR mixing rule and are presented in Table 1. From the measured ion mass concentration and protein concentration, the volume fraction of protein was also calculated (Table 1). The organic contribution was modeled as both BSA and PGM, as both have been used as analogs for respiratory proteins in prior studies (9, 10, 12, 13, 32) (Tables S2 and S3). A comprehensive discussion of the ZSR fitting procedure is described in Section S2. From ZSR fitting, the average *φ*_BSA_ was 0.93 ± 0.01 and 0.93 ± 0.02 in the three lung and trachea samples, respectively. There was no statistical difference in *φ*_BSA_ between the lung and trachea samples (*P* > 0.8). The variance in composition appears to be between individual samples and not correlated to sample collection region.

A method previously employed to relate empirical *φ*_PGM_ measurements with measured GF_90_ was also used here to estimate the fraction of protein in the PRF aerosols (12), which was then compared with the estimates from ZSR fitting. The estimated *φ*_PGM_ from the empirical method (Fig. S1a) are much higher than the *φ*_PGM_ estimated from the ZSR fitting and agree better with the bulk estimates (Table 1).

### Electron microscopy

**Fig. 3.**
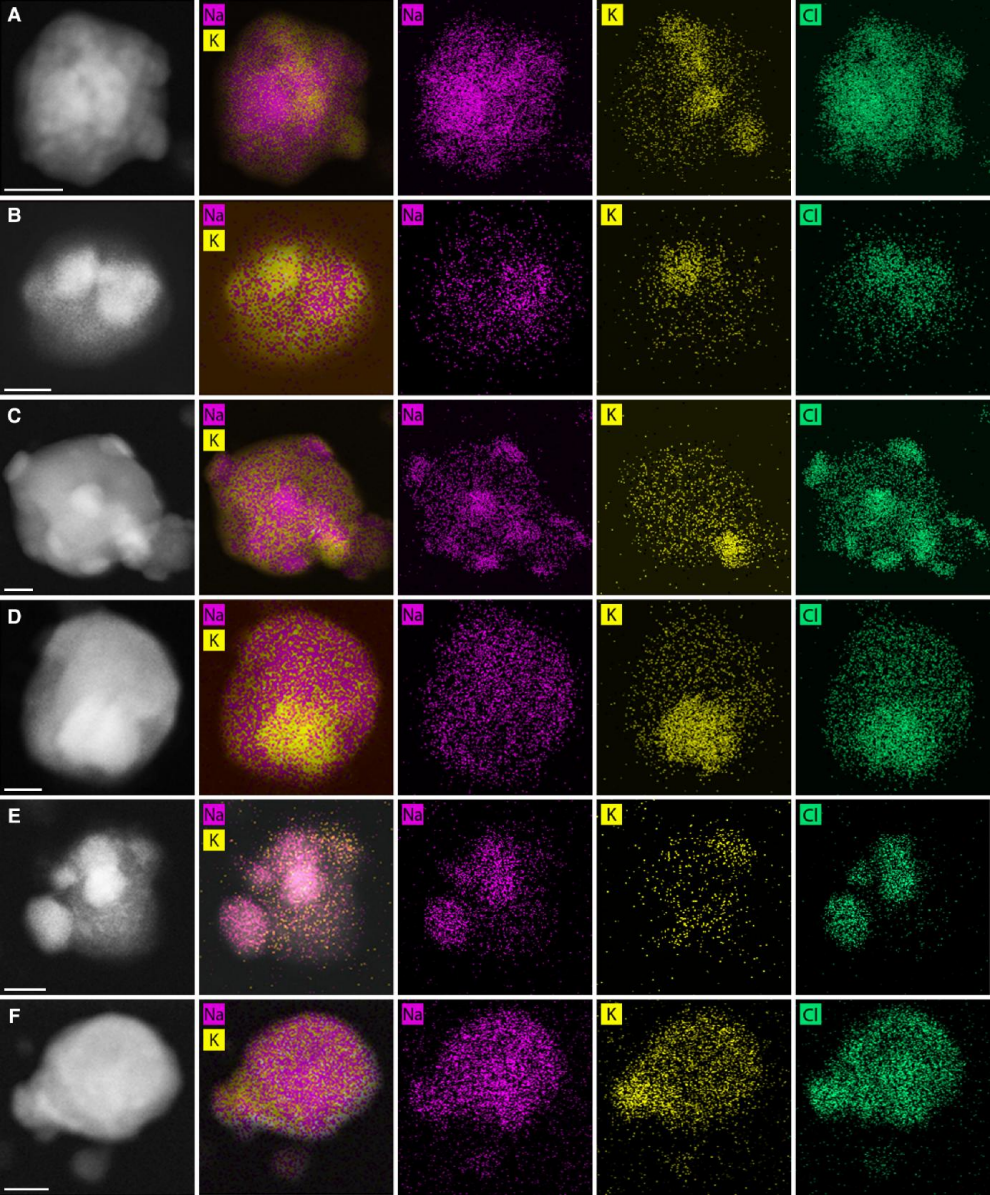
Representative energy-dispersive X-ray spectroscopy (EDS)-mapped images (Na, K, and Cl) of porcine respiratory fluid (PRF) aerosol along with reference dark-field scanning transmission electron micrographs for (A) T3, (B) L3, (C) T5, (D) L5, (E) T6, and (F) L6. The scale bar represents 100 nm.

EDS was used to observe the distribution of Na, K, and Cl throughout PRF and DMEM particles (spectra in Figs. S5 and S6, respectively). Highly concentrated regions of Cl counts indicated a chloride crystal. Crystallinity implied by count clusters was clearly observed in ∼69% of PRF particles observed in EDS (59/85 particles). Crystals could clearly be observed in all DMEM particles. All clustered K and Na regions correspond to clustered Cl regions, suggesting that the primary K- and Na-containing crystals are KCl and NaCl, respectively. Na and K counts were also distributed homogeneously throughout the particles, indicating that there are interactions between the cations and the organics (overlaid Na + K image). Trachea samples (high Na:K) typically included numerous crystals which may be distributed distinctly throughout the particle, indicative of incomplete coalescence of aqueous inorganic phases (Fig. 3A, C, and E). Lung samples (lower Na:K) typically included up to two crystals which were frequently in contact, indicative of complete coalescence of aqueous inorganic phases (Fig. 3B, D, and F). EDS was also performed on DMEM particles for comparison. The large primary crystals contained Na and appeared to be chloride salts from count clustering. It was not clear if K-containing crystals were forming but were certainly less obvious than in PRF aerosol in the EDS-mapped images. Contrary to PRF particles, Ca was abundant and may have formed crystals in DMEM particles. From count clustering, calcium appeared to be associated with phosphorus and may be either crystalline or amorphous phosphates (33).

The morphology of PRF and DMEM aerosol particles was also examined in bright-field electron micrographs (Figs. S3 and S4). Polycrystals can be observed in both PRF and DMEM particles, but in different morphologies. In trachea PRF aerosols (Fig. S4a), as also highlighted in EDS-mapped images, polycrystal morphologies appear as numerous individual crystals which are not necessarily in contact with each other. These numerous individual crystals are embedded in the amorphous organic matrix. The polycrystal DMEM particles (Fig. S4c and e) appear as primarily crystalline, with relatively low amorphicity. These polycrystals appear as numerous crystals nucleated on the surface of a larger crystal, rather than numerous inclusions within an amorphous matrix. Single-crystal inclusions can be observed in DMEM particles (Fig. S4d and f) but are morphologically different to the single inclusions in PRF particles (Fig. S4b). In the example single-crystal PRF micrograph, twinned crystals are in the center of a clearly amorphous matrix. In the DMEM particles (Fig. S4d and f), it is observed that the particle is typically composed of a large crystal with organics coating some of the crystal surface. The primary difference in particle morphology for single-inclusion crystal particles is the volume of amorphous organic (amino acids and dextrose) in the particle.

### Atomic force microscopy

**Fig. 4.**
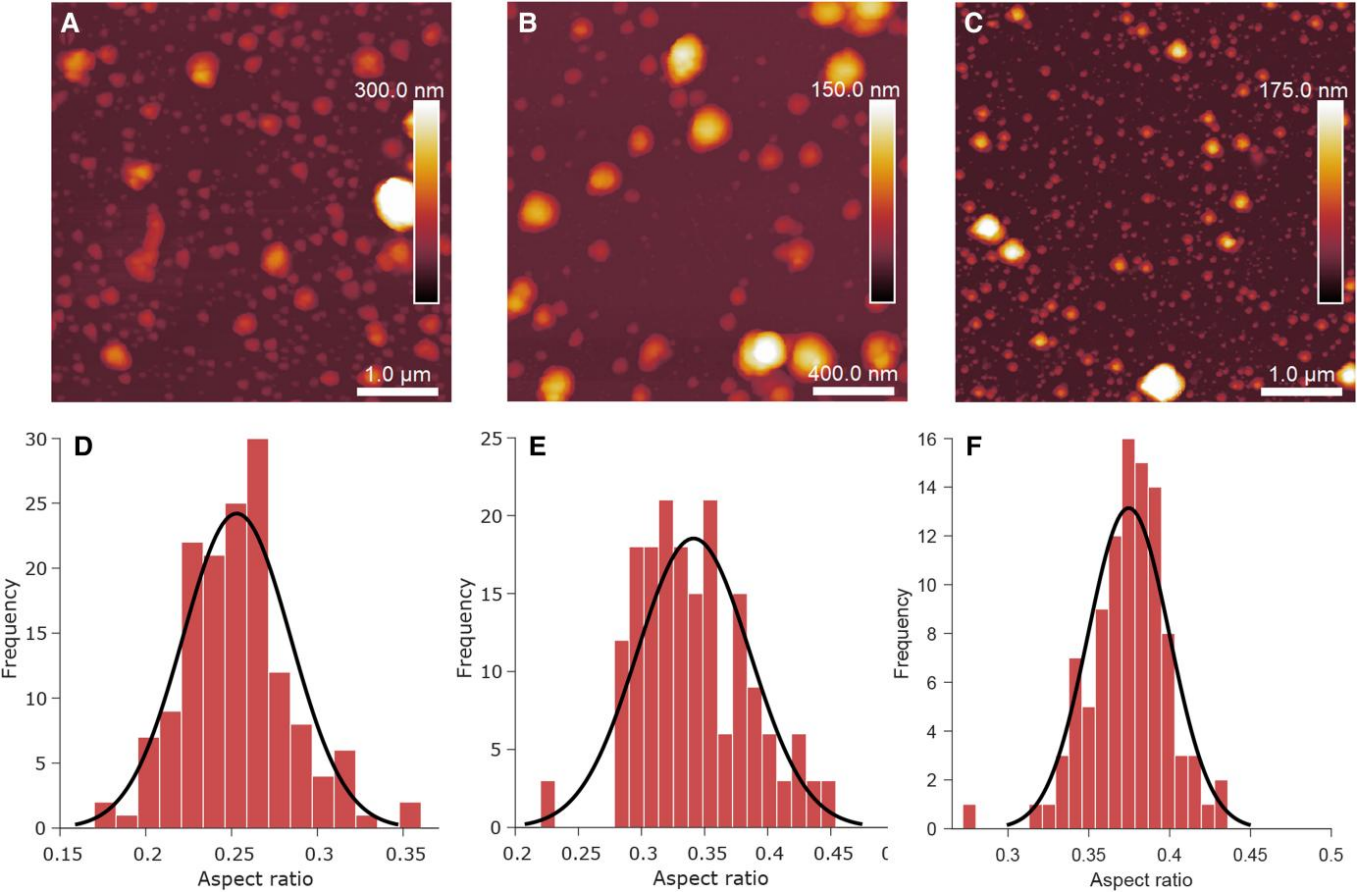
Representative atomic force microscopy (AFM) micrographs (A–C) and aspect ratio (AR) distributions (D–F) and for porcine respiratory fluid (PRF) and DMEM particles. Panels (A) and (D) are PRF lung particles (*N* = 150 particles, sample L6), (B) and (E) are PRF trachea particles (*N* = 177 particles, sample T6), and (C) and (F) are DMEM particles (*N* = 101 particles).

PRF aerosol (*N* = 177 particles, T6, and *N* = 150 particles, L6) and DMEM aerosol (*N* = 101 particles) were collected on Si wafers for AFM analysis (Fig. 4A, B, and C). The AR distribution of aerosol particles can be a useful method to determine the relative phase of particles (solid, semisolid, or liquid) (34) and was measured here (Fig. 4D, E, and F; Section S5). The mean AR measured here for PRF was 0.25 ± 0.03, 0.34 ± 0.04, and 0.37 ± 0.03 for lung (L6), trachea (T6), and DMEM samples, respectively. The AR distributions for PRF lung and trachea samples were found to be statistically different (*P* < 0.001), and the AR distributions for trachea and DMEM samples were also found to be statistically different (*P* < 0.001). The AR for the trachea sample corresponds to the same AR as glucose/NaCl and glucose/MgSO_4_ as reported by Ray et al. (34). The glucose/salt systems were identified as semisolid, and the PRF aerosols measured here are also interpreted as such (AR < 0.365). DMEM, in terms of composition, is approximately a 3:2 mass ratio mixture of NaCl/glucose. As discussed by Ray et al. (34), pure glucose was identified as a semisolid, whereas increasing MgSO_4_ mass concentration in a binary particle increased the AR (more solid-like). Pure MgSO_4_ had a lower AR than pure NaCl, and 1:3 NaCl/glucose had a lower AR than pure NaCl. From this, and from AR > 0.365, it would be expected that a 3:2 NaCl/glucose (approximately DMEM) particle would behave as a solid (34). In prior works, pure protein particles had a mean AR of 0.63 ± 0.01 independent of phase state, whereas systems such as NaCl and glucose had consistent AR–phase correlation (12, 34). Crystals were identified in the TEM micrographs (Figs. S3 and S4), but there were no obvious surface protrusions in the AFM images of PRF particles. This indicates that the crystals are internal features which possibly arise from phase separation between the protein matrix and the aqueous salts.

## Discussion

### Protein volume fraction estimates

The measured *φ*_protein_ from compositional analysis was larger than the modeled value, which leads to three candidate interpretations: (i) the model was not suitable for the experimental data, (ii) the solutes are more hygroscopic than expected, or (iii) the particles were not at a true dry equilibrium (*a*_w_ > 0), and the real GF of the aerosols were larger than measured. For interpretation (i), it is entirely possible that the ZSR mixing rule is not suitable for this simplified system. Physical interactions between solutes, such as surface tension effects of the introduction of amorphous protein phases or unaccounted for surfactants, may influence the diffusion of water through the particle and are not accounted for using the ZSR rule (12). Additionally, incorrect assumptions of the components of the particles will also influence the predicted *φ*_protein_. There may be additional partitioning to the gas phase from carbonates volatilizing into CO_2_ which further reduces particle volume (14). For interpretation (ii), the ZSR rule was evaluated assuming that a highly hygroscopic unknown protein (HUP) accounts for all of the organic component. This evaluation expects the GF at 90% RH (GF_90_) of the HUP must be at least 1.23 ± 0.02. Albumins (35, 36) and mucins (37) have previously been measured in respiratory fluids, indicating that PGM or BSA can be used as surrogates for a fraction of the total protein. Therefore, the HUP must be much more hygroscopic than initially estimated when compared with PGM or BSA, which have GF_90_ equal to 1.11 and 1.07, respectively. There are possibly unaccounted for salts, such as carbonates (14, 36), which may reduce the hygroscopicity of the inorganic fraction. However, this would mean that there would be a greater fraction of inorganics to contribute the same volume of hygroscopicity toward the mixed GF as predicted by the ZSR rule, and it must mean that the unknown inorganics are more hygroscopic than NaCl or KCl, which is unlikely given the lower hygroscopicity of other salts compared with chlorides. For interpretation (iii), proteins may retain strongly bound hydration water, and it is possible that the estimated dry volume fractions assumed at model implementation are underestimated. It is possible that the presence of nonchloride salts (such as sulfates or carbonates) may retain additional water in the form of hydrated salts (14). From these possibilities, the reason for model inaccuracy is likely a combination of oversimplification of solutes and additional physical processes which affect the measured size of the particles.

In a previous study, the *φ*_PGM_ was determined in a bulk solution and compared with the particles’ GF_90_ from which a relationship was determined (12). The aerosol composition in the PRF is expected to be similar to that used in the previous study, and that technique was adapted here. This method shows better agreement to the *φ*_protein_ measured in bulk here (Fig. S1; Table 1), although it is not certain that this method is accurate for complex mixed systems like PRF.

### Crystal formation in dried respiratory aerosols

**Fig. 5.**
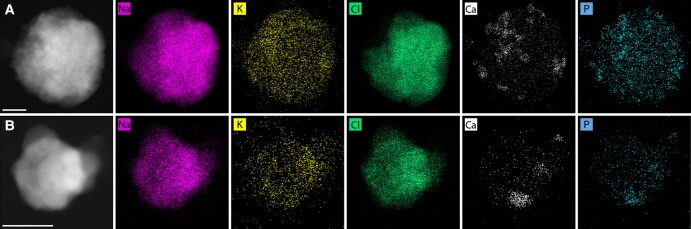
Energy-dispersive X-ray spectroscopy (EDS)-mapped images (Na, K, Cl, Ca, and P) of DMEM aerosol along with reference dark-field scanning transmission electron micrographs. The scale bar represents 500 nm.

The EDS maps (Figs. 3 and 5) show good agreement between Na/K clusters and Cl clusters, indicating that Na^+^ and K^+^ ions formed salts with Cl^−^ ions (NaCl and KCl). An example of validation of a NaCl crystal phase in a DMEM particle is shown in Fig. S7 (38). It was typically observed that there were more numerous crystals in the trachea particles, which had a higher ratio of Na:K measured in the bulk. As discussed in prior publications, the crystal nucleation behavior of mixed organic/inorganic aerosol is influenced by the drying rate of the particles and the viscosity of the organic matrix (11, 12). At high RH (e.g. immediately at generation), the particle is a homogeneous liquid mixture of organic and inorganics, and as it loses water through dehydration, the organic/inorganic phases separate. If the inorganics separate into numerous aqueous inclusions, giving sufficient equilibration time at constant conditions, the numerous inclusions will coalesce into a single inclusion. That single inclusion then corresponds to a single crystal upon efflorescence. The difference in crystal nucleation behavior between the lung and trachea samples can then be explained as a consistent difference in either the organic matrix or the separation of the inorganic phase. There is no statistically significant difference in the measured total protein concentration between the trachea and lung samples here; however, research has reported that the concentration and surface activity of surfactants are lower in the trachea (39). There may be increased surface tension at phase interfaces within the trachea particles which may limit microstructural rearrangement of the inorganic inclusions between organic layers, promoting the formation of multiple crystals.

The morphology of DMEM aerosols was similar to that of PRF aerosols only in shape. DMEM aerosols appeared to contain large crystals which dominated particle volume, whereas regions of amorphous organics are much clearer in PRF aerosols. These results indicate that PRF, compared with DMEM, had considerably more potassium and protein and considerably less carbonate and calcium. These compositional differences affected the morphology and crystal formation in the particles. The dominant crystal in both PRF and DMEM appeared to be NaCl; however, PRF also contained numerous KCl crystals, which was not clearly observed in DMEM aerosols. DMEM particles contained calcium crystals, whereas PRF particles did not. These differences in crystal formation and composition would cause different pathogen microenvironments during evaporation, which would influence virus viability in unknown ways. PRF particles seemed to have consistent crystal habits, in contrast with other works (9).

### Implications for virus viability and airborne transmission

The measured AR distribution of the PRF trachea sample shows similarity to the AR measured in salts mixed with viscous organics in previous measurements, whereas the lung sample had AR more similar to glutaric acid (34). The authors demonstrated a relationship between AR and particle phase state, which would indicate that the effloresced trachea PRF particles measured here are in a semisolid phase (AR < 0.365) and, within the uncertainty, may be in a solid phase. The AR for the dried lung PRF sample (AR < 0.365) indicates that they are semisolid. Dried DMEM particles were expected to be in a solid state (AR > 0.365). Previous works have hypothesized a connection between the viscosity of semisolid organic phases and the viability of embedded viruses, although this remains experimentally inconclusive (9, 20, 40).

It has been demonstrated that in evaporating simulated respiratory drops, a surfactant (dipalmitoylphosphatidylcholine, DPPC) was partitioned to the organic–inorganic interface rather than at the air–particle interface (10). As discussed earlier, the location of the virus may influence the viability. In this study, it was demonstrated that lung and trachea aerosols had statistically different Na:K ratio, *φ*_protein_, and AR. Trachea fluid had a higher Na:K ratio, larger AR, and a higher protein fraction than lung fluid. Given the GF_90_ are similar between all samples, this indicates that higher fractions of NaCl compared with KCl may accommodate higher organic fractions while retaining the same GF, as NaCl is more hygroscopic than KCl. Importantly, the crystal nucleation within trachea particles was different to that observed in lung particles. Trachea particles had numerous crystals dispersed throughout the particles, which indicates that prior to efflorescing, there were numerous aqueous salt inclusions which had not completely coalesced into a single inclusion (11). If the surfactant is partitioned to the organic–inorganic interface, as discussed by Vejerano and Marr (10), then the number of aqueous salt inclusions becomes important. For particles with numerous small inclusions, there is greater interfacial surface area for the surfactant to cover. Enveloped viruses, which are covered with a lipid membrane, will likely partition to the regions of the respiratory particle which contains lipids, such as surfactants, and therefore will partition to the organic–inorganic interface. If the surfactant prevents the viral envelope from degradation, then particles with fewer aqueous inclusions (such as the lung PRF samples discussed earlier) may be favorable for virus viability due to lower interfacial surface area between the aqueous salts. The disinfectant effect of concentrated aqueous salts by denaturing surface proteins is commonly assumed to be a primary mechanism of viral inactivation. This disinfectant effect may be more prominent in particles with numerous aqueous salt inclusions. The combined inactivation effects of different salts (such as NaCl and KCl) are also currently not well understood and may also be influenced by the number of aqueous inclusions. This would indicate that particles with smaller interfacial surface area would be favorable for virus viability, as viruses could partition through the surfactant into the inorganic phase. Alternatively, in particles which are weakly hydrated at low RH (which may include proteins), there may be higher alkalinity associated with the organic fraction due to the persistence of ${\mathrm{CO}}_{3}^{2\text{-}}$ from the volatilization of CO_2_ from the particles. DMEM likely contains a much higher concentration of carbonate than PRF and therefore would be more alkaline than respiratory fluid. X-ray crystallography of DMEM aerosol data suggests that under ambient conditions the only crystal present in DMEM is NaCl (Fig. S8). There is strong evidence for the presence of ${\mathrm{HCO}}_{3}^{\text{-}}$ in the infrared absorbance spectrum of dried DMEM aerosol (Fig. S10). There is also no evidence for the presence of free liquid water or ${\mathrm{CO}}_{3}^{2\text{-}}$ (Section S7). This indicates that on the timescale of evaporation, ${\mathrm{HCO}}_{3}^{\text{-}}$ was not converted to ${\mathrm{CO}}_{3}^{2\text{-}}$ through the volatilization of CO_2_. The lack of liquid water and carbonate indicates that once the DMEM particles have dried, there is a cessation of chemical processes under stable ambient conditions, which prevents the basification of the particles. This assumption is not valid for particles which remain above the efflorescence RH of the salt species within the droplet.

The collection method of the PRF may serve as a limitation in this study. The respiratory fluid was diluted during collection in water, which may affect the composition of the recovered fluid by preferentially recovering more water-soluble components. However, because the primary component of respiratory fluid is already water, this will likely not influence results. Secondly, the method of particle generation may influence the composition and size of the particles. Specifically, it has been extensively demonstrated that particles generated using Collison-type nebulizers do not adequately represent the modality of sea spray aerosol (SSA), which exhibits similar production method and modality to respiratory particles (41–43). The modality of Collison-generated particles is typically unimodal or bimodal, depending on exact configuration, whereas respiratory aerosols can exhibit numerous modes (42, 44). The count median diameter (CMD) of human cough aerosol in our previous study was measured using a scanning mobility particle sizer to be ∼100 nm, which informed the choice for using a preclassified diameter of 100 nm for the TDMA work in this study (20). It is possible that there was organic enrichment in the smaller particles studied here, but it would be expected that this enrichment would also be present in human expiratory aerosol (26, 42, 45–47). Whether the composition of human respiratory aerosol is the same as bulk respiratory fluid, and whether the composition is size-dependent, is an open and critical research question. Finally, the drying rate of the particles in this study may be quicker than what would be experienced in an expiratory plume (48). Particles in a plume will remain entrained for some time during transport and, thus, will experience a period of equilibration in the warm and humid plume before mixing with the ambient air and equilibrating further. The method employed in this work did not have this initial equilibration and therefore may have reached a different resulting morphology. The high vacuum environment of the TEM is also dissimilar to the ambient conditions of the indoor environment but was unlikely to impact the morphology of already dried particles (12).

The physicochemical properties of PRF from the trachea and lungs were investigated and compared with growth media (DMEM). Na and K were the metals measured with the highest concentration in both PRF and DMEM, but the mass ratio of Na to K ions in DMEM was significantly higher than that in PRF. There was also a statistically significant difference in Na:K between lung and trachea samples, indicating that the composition of respiratory aerosol differs between production region. Chloride crystals (both Na and K) were frequently observed in PRF aerosol, but DMEM aerosols were Na-dominant crystals. The ratio of Na:K in PRF was linked to a difference in crystal nucleation and particle morphology upon evaporation. The crystals frequently appeared as separated individual crystals, indicating that the aqueous salts did not fully coalesce prior to efflorescence. This is indicative of rapid drying times and a highly viscous organic matrix, which are not sufficient to equilibrate the particle with the ambient atmosphere. It may be unfavorable for viruses which are partitioned to the organic–inorganic interface in particles which have numerous aqueous salt inclusions. Due to greater interfacial surface area, surfactants at the organic–inorganic interface may not sufficiently cover the entire inclusion and may not provide protection for the viruses. The hygroscopicity of PRF is similar to that of HRF, indicating that the compositions of PRF and HRF are similar and PRF may be a suitable analog for HRF in aerovirology studies. The carbonate concentration of PRF and DMEM aerosols was estimated, and PRF was likely less alkaline than DMEM. The volume fraction of proteins measured in the PRF was >97% in all samples (∼93% protein by dry solute mass fraction). This indicates that to accurately represent the microenvironment experienced by an expired pathogen, sufficiently high protein content must be used. DMEM had considerably lower potassium and protein content than PRF, indicating that the pathogen microenvironment provided by growth media aerosols is not representative of a real-world virus expiration scenario. Further care must be taken in aerovirology studies to ensure that the ionic composition and the protein content of the virus suspension are adequately representative of respiratory fluid, as we demonstrated here that the morphology and crystallinity of respiratory particles are highly dependent on composition.

## Supplementary Material

pgad087_Supplementary_Data

## Data Availability

All data that were analyzed in this study are available in the main manuscript or are available in the supplementary information.
