# Supplementary material for: Physicochemical characterization of porcine respiratory aerosol and considerations for future aerovirology
Source: PNAS Nexus. 2023 Mar 22;2(3):pgad087. doi: 10.1093/pnasnexus/pgad087 (PMC10063220; doi:10.1093/pnasnexus/pgad087)
Supplement: pgad087_Supplementary_Data [file pgad087_supplementary_data.docx]

**Physicochemical characterization of porcine respiratory aerosol and considerations for future aerovirology**

Robert Groth^1^, Sadegh Niazi^1^, Kirsten Spann^2^, Graham R. Johnson^1^, and Zoran Ristovski^1*^

^1^School of Earth and Atmospheric Sciences, International Laboratory for Air Quality and Health, Faculty of Science, Queensland University of Technology, Brisbane, QLD 4000, Australia

^2^School of Biomedical Sciences, Centre for Immunology and Infection Control, Faculty of Health, Queensland University of Technology, Brisbane, QLD 4000, Australia

*Corresponding author: Zoran Ristovski, z.ristovski@qut.edu.au

This supplementary manuscript contains 13 pages.

Figures S1 – S10

Tables S1 – S3

## **S1. Compositional measurements of porcine respiratory fluid**

| Sample site/ID | Na (g/L) | K (g/L) | Ca (g/L) | Na:K | Total protein (g/L) |
| --- | --- | --- | --- | --- | --- |
| L1 | 0.1426 | 0.0855 | 0.0077 | 1.67 | 9.75 |
| T1 | 0.3217 | 0.0936 | 0.0170 | 3.44 | 12.88 |
| L2 | 0.0595 | 0.0288 | 0.0034 | 2.07 | 12.26 |
| T2 | 0.0802 | 0.0306 | 0.0080 | 2.62 | 9.75 |
| L3 | 0.1648 | 0.0599 | 0.0045 | 2.75 | 11.22 |
| T3 | 0.1139 | 0.0354 | 0.0061 | 3.22 | 9.75 |
| L4 | 0.0646 | 0.0318 | 0.0039 | 2.03 | 12.26 |
| T4 | 0.0985 | 0.0392 | 0.0050 | 2.51 | 10.65 |
| L5 | 0.1236 | 0.0509 | 0.0061 | 2.43 | 11.22 |
| T5 | 0.0760 | 0.0299 | 0.0047 | 2.54 | 12.91 |
| L6 | 0.0753 | 0.0400 | 0.0051 | 1.88 | 12.47 |
| T6 | 0.0830 | 0.0250 | 0.0089 | 3.32 | 12.88 |

**Table S1.** Porcine respiratory fluid (PRF) lung (L) and trachea (T) samples for compositional analysis. Elemental composition (Na, K, and Ca) was analysed using inductively coupled plasma optical emission spectroscopy (ICP-OES), and protein composition was measured using a BCA Protein Assay kit. The components of each sample are diluted by an unknown amount, and so the mass ratio is more useful for comparison between samples.


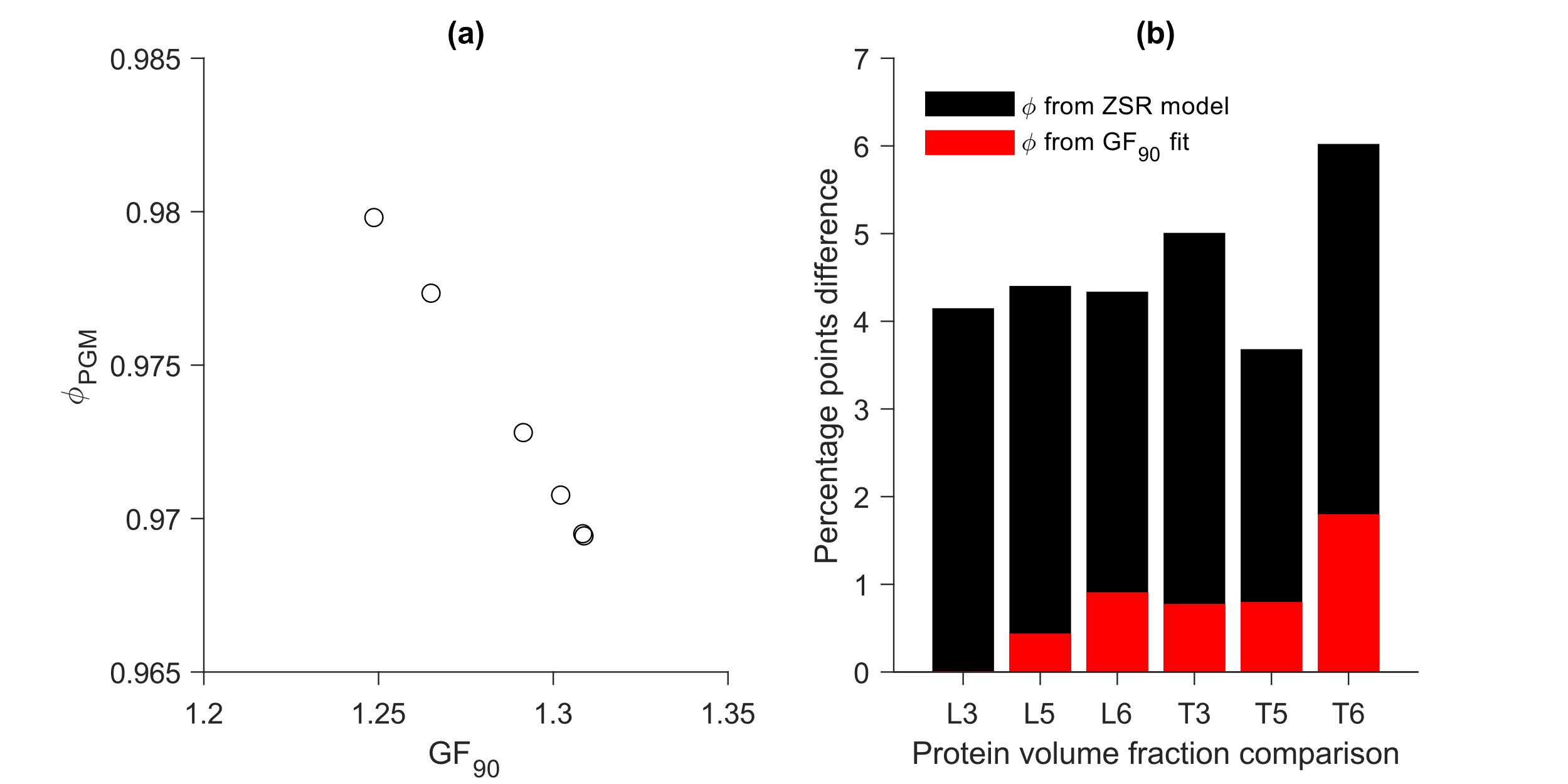


**Figure S1.** (a) Estimated organic volume fraction of porcine gastric mucin (PGM) in aerosols ($\phi_{PGM}$) as a function of diametric growth factor at 90% relative humidity (GF_90_) (1), (b) comparison between ZSR fitted $\phi_{PGM}$ and empirically estimated $\phi_{PGM}$ when compared to measured $\phi_{protein}$ (main manuscript, Table 1).


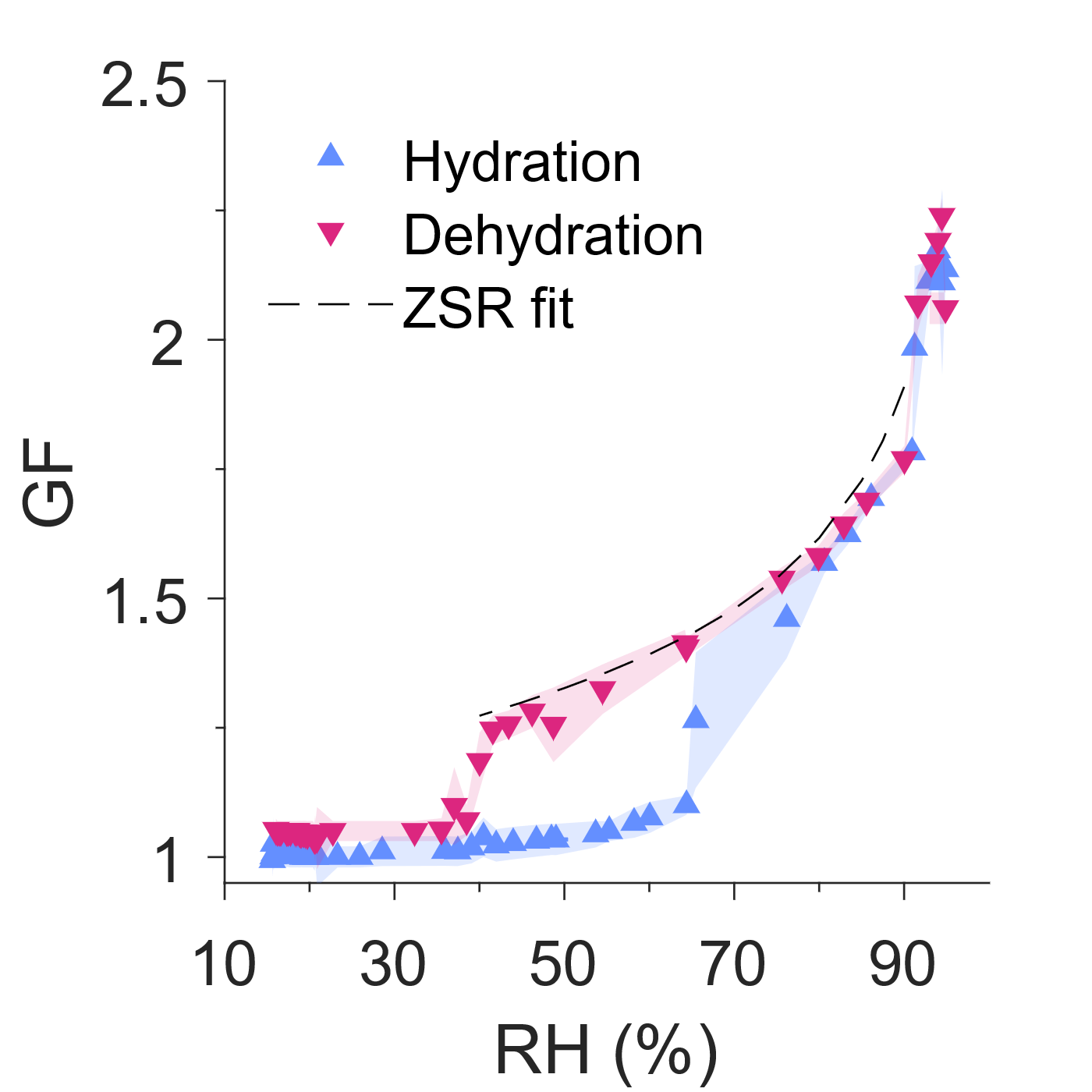


**Figure S2.** Diametric hygroscopic growth (GF) measurements of DMEM aerosol as measured by humidification tandem differential mobility analyser (H-TDMA).

## **S2. Compositional estimates of porcine respiratory fluid from modelling**

The organic volume fraction ($\phi_{org}$) of the porcine respiratory fluid (PRF) aerosols were estimated using the Zdanovskii-Stokes-Robinson (ZSR) mixing rule (Equation S1).

$$\begin{aligned} GF_{\mathrm{mix}}^{3}=\sum_{i}^{n} GF_{i}^{3}\phi_{i}\#\left( 1 \right) \end{aligned}$$

$$\begin{aligned} GF_{\mathrm{mix}}^{3}=GF_{\mathrm{NaCl}}^{3}\phi_{\mathrm{NaCl}}+GF_{\mathrm{KCl}}^{3}\phi_{\mathrm{KCl}}+GF_{\mathrm{org}}^{3}\phi_{\mathrm{org}}\#\left( 2 \right) \end{aligned}$$

$$\begin{aligned} \phi_{\mathrm{org}}=\frac{GF_{\mathrm{mix}}^{3}}{GF_{\mathrm{NaCl}}^{3}\phi_{\mathrm{NaCl}}+GF_{\mathrm{KCl}}^{3}\phi_{\mathrm{KCl}}+GF_{\mathrm{org}}^{3}}\#\left( 3 \right) \end{aligned}$$

The solute volume fraction of NaCl ($\phi_{NaCl}$) and KCl ($\phi_{KCl}$) are not known, but expressions for these quantities can be derived from the cation mass ratio $R_{i}$ and $\phi_{org}$.

$$\begin{aligned} R_{i}=\frac{m_{Na}}{m_{K}}\#\left( 4 \right) \end{aligned}$$

First, the solute volume fraction of salts ($\phi_{salt}$) can be calculated from $\phi_{org}$, and then expressed in terms of the volume of each component and the total particle volume ($V_{p}$).

$$\begin{aligned} \phi_{salt}=1-\phi_{org}\#\left( 5 \right) \end{aligned}$$

$$\begin{aligned} \phi_{salt}=\frac{V_{NaCl}+V_{KCl}}{V_{p}}\#\left( 6 \right) \end{aligned}$$

For a particle of arbitrary volume $V_{p}=1$, Equation S6 is then reduced to

$$\begin{aligned} \phi_{salt}=V_{NaCl}+V_{KCl}\#\left( 7 \right) \end{aligned}$$

The volume of the salts can then be expressed through the fundamental density relation as

$$\begin{aligned} \phi_{salt}=\frac{m_{NaCl}}{\rho_{NaCl}}+\frac{m_{KCl}}{\rho_{KCl}}\#\left( 8 \right) \end{aligned}$$

where $m_{i}$ are masses of salt $i$ and $\rho_{i}$ are densities of salt $i$. An additional equation is needed to solve the two unknowns ($m_{NaCl}$ and $m_{KCl}$), so the masses of the salts may be related through $R_{i}$, the number of moles $n_{i}$ of species $i$, and the molar masses $M_{i}$ of species $i$ as Equation S13.

$$\begin{aligned} m_{NaCl}=n_{NaCl}M_{NaCl}=n_{Na}M_{NaCl}\#\left( 9 \right) \end{aligned}$$

$$\begin{aligned} m_{KCl}=n_{KCl}M_{KCl}=n_{K}M_{KCl}\#\left( 10 \right) \end{aligned}$$

$$\begin{aligned} n_{Na}=\frac{m_{Na}}{M_{Na}}\#\left( 11 \right) \end{aligned}$$

$$\begin{aligned} n_{K}=\frac{m_{K}}{M_{K}}\#\left( 12 \right) \end{aligned}$$

$$\begin{aligned} \frac{m_{NaCl}}{m_{KCl}}=\frac{n_{Na}M_{NaCl}}{n_{K}M_{KCl}}=\frac{m_{Na}M_{K}M_{NaCl}}{m_{K}M_{Na}M_{KCl}}=R_{i}\frac{M_{K}M_{NaCl}}{M_{Na}M_{KCl}}\#\left( 13 \right) \end{aligned}$$

Then, expressing Equation S13 in terms of $m_{NaCl}$,

$$\begin{aligned} m_{NaCl}=m_{KCl}R_{i}\frac{M_{K}M_{NaCl}}{M_{Na}M_{KCl}}\#\left( 14 \right) \end{aligned}$$

and inserting Equation S14 into Equation S8, we arrive at

$$\begin{aligned} \phi_{salt}=\frac{m_{KCl}}{\rho_{KCl}}+\frac{1}{\rho_{NaCl}}m_{KCl}R_{i}\frac{M_{K}M_{NaCl}}{M_{Na}M_{KCl}}\#\left( 15 \right) \end{aligned}$$

Then, rearranging Equation S15 for $m_{KCl}$ and consolidating density and molar mass into molar volume $\bar{V_{i}}$ of species $i$, we get

$$\begin{aligned} m_{KCl}=\frac{{\rho_{KCl}\phi}_{salt}}{1+R_{i}\frac{M_{K}\bar{V}_{NaCl}}{{M_{Na}\bar{V}}_{KCl}}}\#\left( 16 \right) \end{aligned}$$

The mass of KCl can be equated from Equation S16 and then inserted into Equation S14 to calculate the mass of NaCl. The mass of the salts can then be converted to volumes using the fundamental density relation, and then expressed as $\phi_{NaCl}$ and $\phi_{KCl}$ and used with Equation S3 to calculate $\phi_{org}$ of the aerosol samples. Prior to further analysis, the calculated masses must be appropriately scaled to the real particle volume which was assumed to be 1 in Equation S6.

The diametric hygroscopic growth factor (GF) of NaCl and KCl was calculated using the AIOMFAC model (https://aiomfac.lab.mcgill.ca) (1, 2). For the organic components of the aerosols, both porcine gastric mucin (PGM) and bovine serum albumin (BSA) were considered as analogues, as both have characterized hygroscopic growth and have been used in previous studies (3–7). The GF of PGM was calculated from Equation S17 (4) and the GF of BSA was calculated from Equation S18 (6, 7), where $\alpha_{i}$ and $\beta_{i}$ are polynomial coefficients, and $S=\frac{RH}{100}$. The polynomial coefficients for Equation S17 and Equation S18 are in Table S2 and Table S3, respectively.

$$\begin{aligned} GF_{\mathrm{PGM}}=\left( 1+\left( \alpha_{0}+\alpha_{1}S+ \alpha_{2}S^{2} \right)\frac{S}{1-S} \right)^{\frac{1}{3}}\#\left( 17 \right) \end{aligned}$$

| Polynomial coefficient | $\alpha_{0}$ | $\alpha_{1}$ | $\alpha_{2}$ |
| --- | --- | --- | --- |
| Value | 0.0355 | 0.10516 | -0.10976 |

**Table S2.** Polynomial coefficients for calculating the diametric growth factor (GF) of porcine gastric mucin (PGM) aerosol particles (Equation S17).

The fit form presented in Equation S17 was refit from the data presented by Groth et al. to be in the same polynomial form as presented by Pöhlker et al. (4, 7).

$$\begin{aligned} GF_{\mathrm{BSA}}=\left( 1+\left( \beta_{0}+\beta_{1}S+ \beta_{2}S^{2} \right)\frac{S}{1-S} \right)^{\frac{1}{3}}\#\left( 18 \right) \end{aligned}$$

| Polynomial coefficient | $\beta_{0}$ | $\beta_{1}$ | $\beta_{2}$ |
| --- | --- | --- | --- |
| Value | 0.111 | 0.0239 | -0.131 |

**Table S3.** Polynomial coefficients for calculating the diametric growth factor (GF) of bovine serum albumin aerosol particles (Equation S18).

The best fit value for $\phi_{org}$, considering both PGM and BSA as the candidate organic component, was then calculated from the experimental H-TDMA data (for $GF_{mix}$) and ICP-OES data (for $R_{i}$) and determined via ordinary least squares (OLS) minimization.

## **S3. Bright-field transmission electron micrographs**

Low-magnification transmission electron micrographs were taken of the samples detailed in the main manuscript (L3, T3, L5, T5, L6, T6) to observe general particle morphology. When the sample is below eucentric height (under focused), Fresnel fringes are visible as a white outline surrounding a particle. If a crystalline region is strongly diffracting and below eucentric height, it will appear as a black spot (the crystal) and a white spot with equivalent geometry (the diffracting electrons). This can be useful for observing particle crystallinity for a collection of nanoparticles, and an example of this is circled in red in Figure S3b.


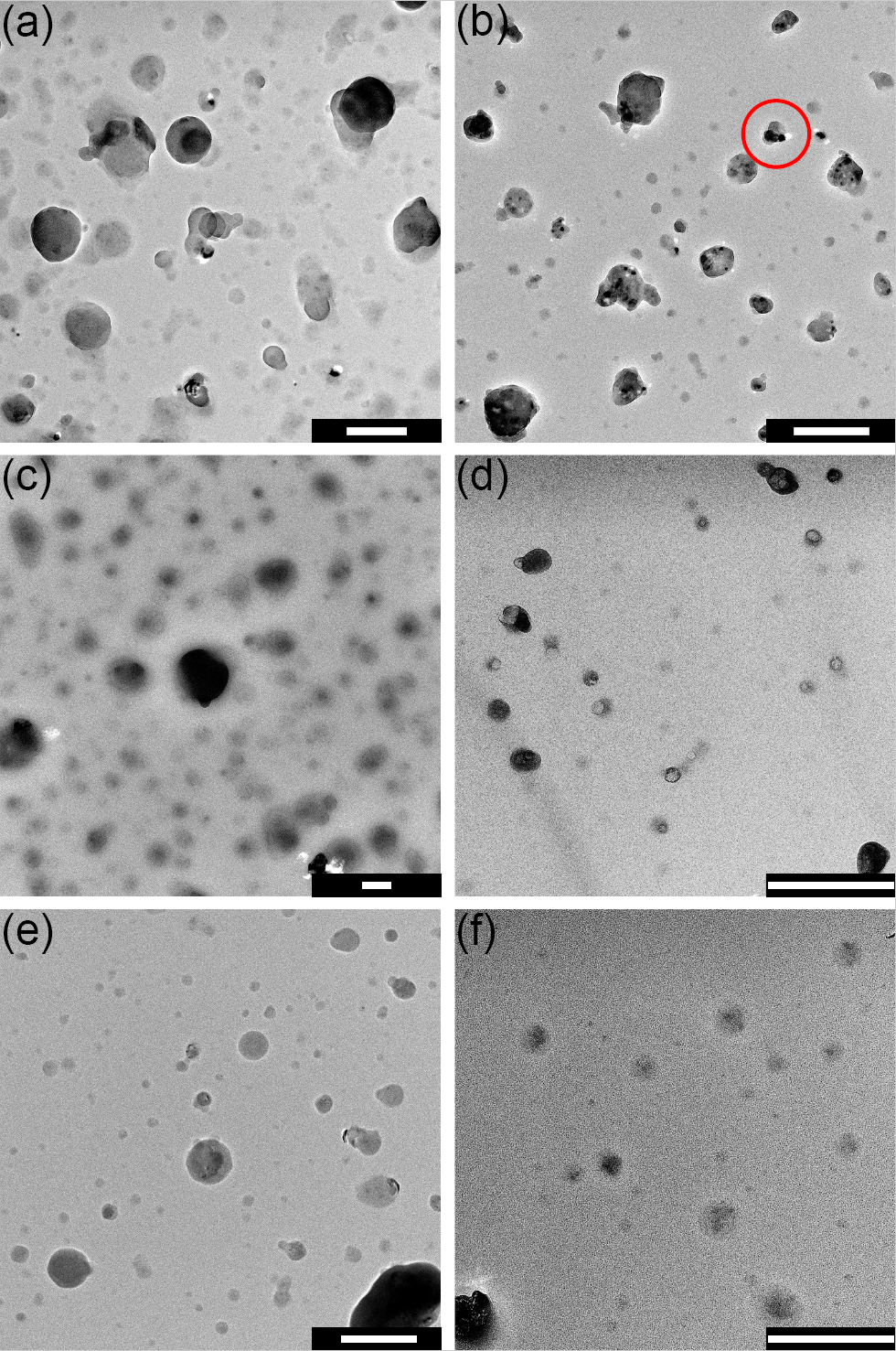


**Figure S3.** Low-magnification transmission electron micrographs (LM-TEM) for samples (a) L3, (b) T3, (c) L5, (d) T5, (e) L6, and (f) T6. The scalebar represents 500 nm.


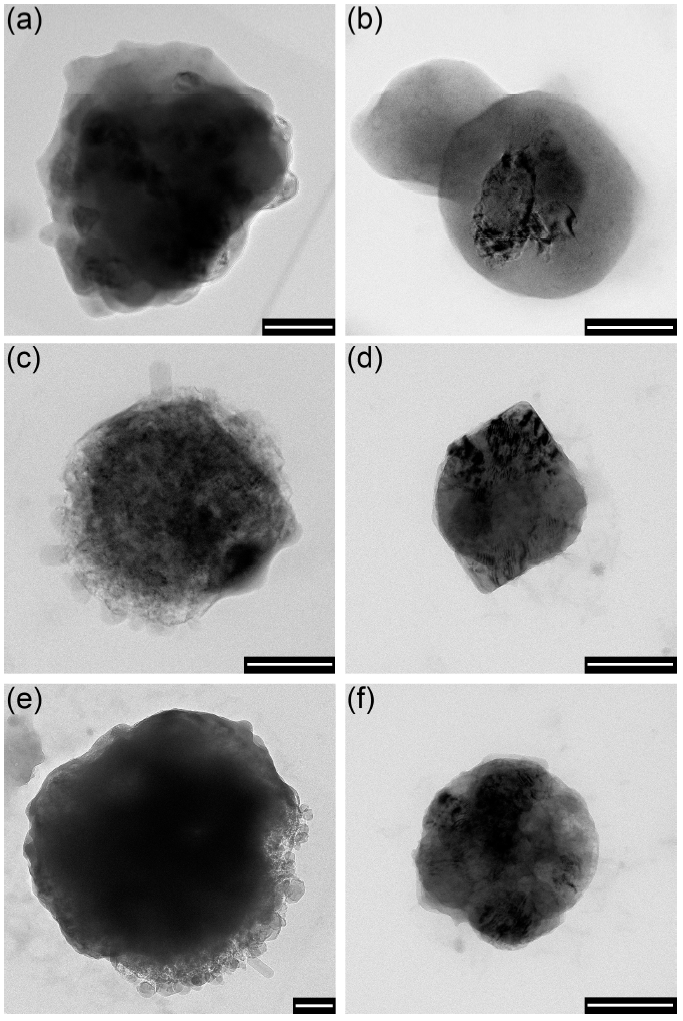


**Figure S4.** Transmission electron micrographs of porcine respiratory fluid (PRF) (a) trachea and (b) lung particles. Panels (c) - (f) are DMEM particles. The scalebar represents 200 nm.

## **S4. Elemental analysis from energy-dispersive X-ray spectroscopy**

Energy-dispersive X-ray spectroscopy (EDS) was used to determine the localization of chloride crystals within PRF aerosol particles. Representative EDS map images are in the main manuscript, and here, the spectra are presented for those EDS map images. Several elements are not analytically useful due to their presence within the sample environment. O and C are present within the Formvar coating of the TEM grid, the support bars of the TEM grid are composed of Cu, and several EDS detector components are made from Si. Na, K, Cl, S, and P are all therefore expected to be contributed entirely from the PRF samples. Ca was not detected in significant concentrations to contribute to the spectra. The spectral peaks of analytical interest are sodium K_α_, potassium, K_α_ & K_β_, chlorine K_α_ & K_β_, phosphorus K_α_ & K_β_, sulfur K_α_ & K_β_, and calcium K_α_ & K_β_ energies. The copper K_α_ & K_β_ energies are useful references to determine the total exposure between samples, as the only contribution of Cu is expected to be from the TEM grid bars.


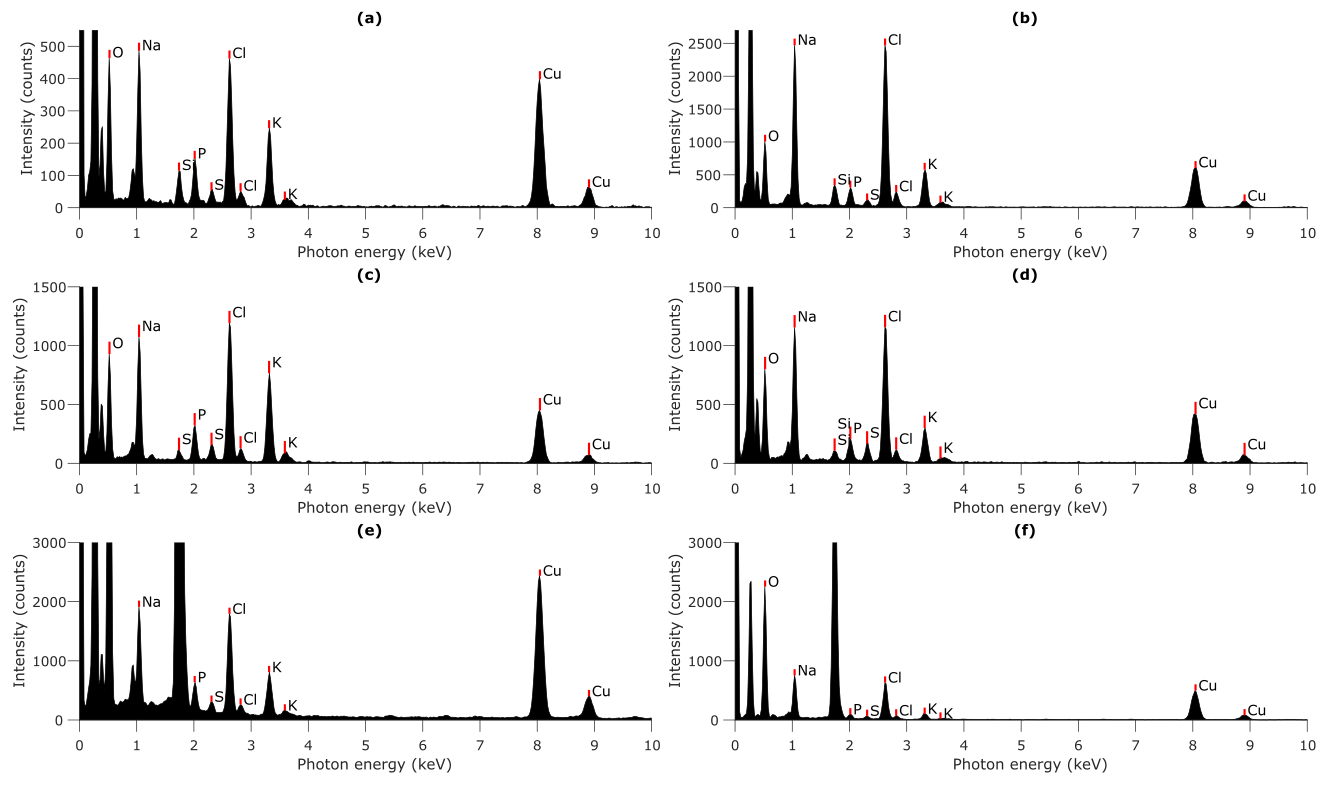


**Figure S5.** Annotated energy-dispersive X-ray spectra of porcine respiratory fluid (PRF) aerosol samples (a) L3, (b) T3, (c) L5, (d) T5, (e) L6, and (f) T6.


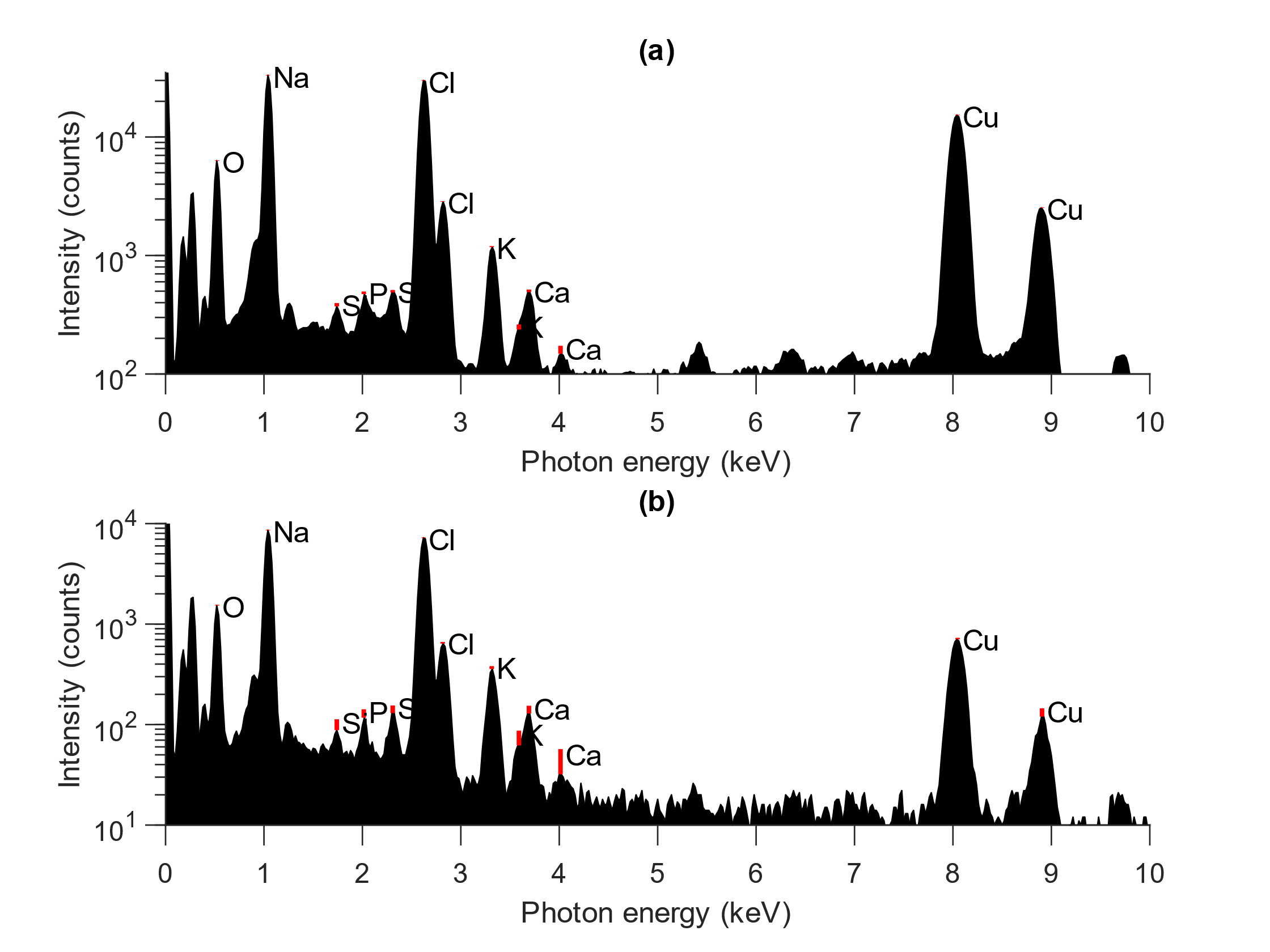


**Figure S6.** Annotated energy-dispersive X-ray spectra of DMEM aerosol.

The spatial distribution of elements in the EDS maps of one DMEM sample were analysed by method of cluster analysis (8). The method of Münch et al. was performed using k-means clustering and predefined centre clustering. The predefined centres, based from EDS maps, diffraction, and the known composition of DMEM, were: NaCl, KCl, Na_2_CO_3_, CaCO_3_, NaHCO_3_, KHCO_3_, CaHPO_4_, and Ca(H_2_PO4)_2_. In this particular case, the EDS maps (main text, Figure 4a) suggest the presence of a large NaCl crystal comprising much of the bulk o the particle. This is due dense counts of Na clustered with Cl in an irregular shape. On the right of the irregular shape, higher density of K, Ca, and P counts are observed concurrent with low/no Na/Cl counts, suggesting that this may be the boundary of an NaCl cluster. The cluster-analysed image (Figure S7a,b) reinforces this with a NaCl (halite) phase comprising most of the particle with the presence of other mineral phases being preferred over NaCl in other regions of the particle. This approach, however, has several key limitations. Notably, the particle is expected to have crystals in 3-dimensions, contrary to a thin-section of a mineral sample which has large and well-defined grain boundaries (8). This means that while it is unlikely that the regions of the particle which have less-dense Na/Cl counts contain NaCl crystals, it could instead be that these regions have a thin NaCl crystal with other crystals on top or below, or that increased C counts from the organic phase instead cause the algorithm to prefer carbonate or phosphate species, as highlighted by the contribution of additional elements in the pseudo phase spectra (Figure S7a). This approach on its own is not suitable for these types of particles for determining the presence of small crystal phases, however, in combination with selected-area electron diffraction (SAED, Figure S9), it becomes clear that a DMEM particle can contain one large NaCl region with several amorphous, or non-NaCl, regions.


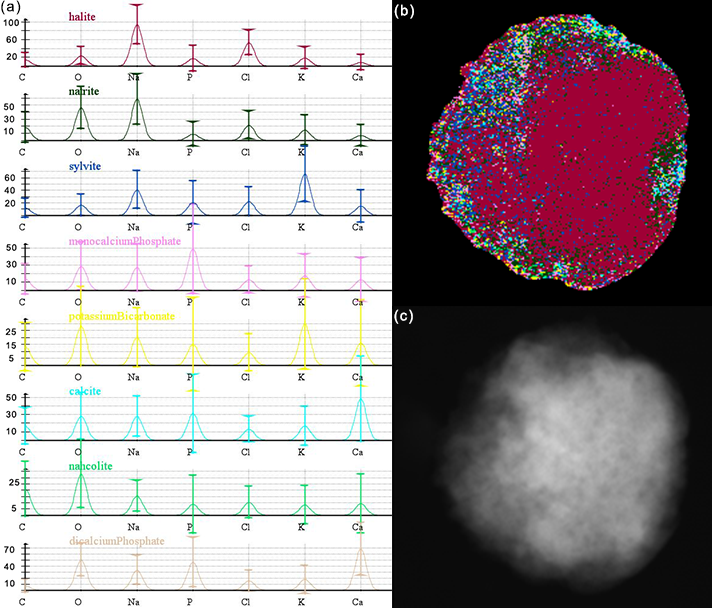


**Figure S7.** Predefined centre clusters of one DMEM particle. The (a) predefined centres were informed by k-means clustering of the elemental maps and were (b) mapped onto the (c) STEM image.

## **S5. Atomic force microscopy**

Porcine respiratory fluid (PRF, samples T6 and L6) and DMEM aerosol particles were deposited onto a Si wafer substrate. These particles then deform according to their momentum and viscoelastic properties. The AFM micrographs were plane flattened prior to analysis. The aspect ratio (AR) is the ratio of the maximum particle height ($H_{M}$) to the particle area equivalent diameter ($D_{\mathrm{AE}}$), as defined in Equation S19.

$$\begin{aligned} \mathrm{AR}=\frac{H_{M}}{D_{\mathrm{AE}}}\#\left( 19 \right) \end{aligned}$$

The area equivalent diameter is defined as the diameter of a circle with equivalent area to the area of the particle (Equation S20).

$$\begin{aligned} D_{\mathrm{AE}}=2\sqrt{\frac{A}{\pi}}\#\left( 20 \right) \end{aligned}$$

In the case of this study, the particle area $A$ was obtained from the NanoScope Analysis software used to interpret the AFM data.

## **S6. Crystallography**

Dried DMEM particles were electrostatically deposited onto a (522) Si wafer for several hours to produce a film. The crystallinity of particles in the film was measured using grazing incidence diffraction (GID) was performed using a Rigaku SmartLab X-ray diffractometer, using Cu-K_α_ X-rays (40 kV, 40 mA) with a downstream 0.228 degree parallel slit analyser. Crystal phases were identified using X’Pert HighScore Plus.


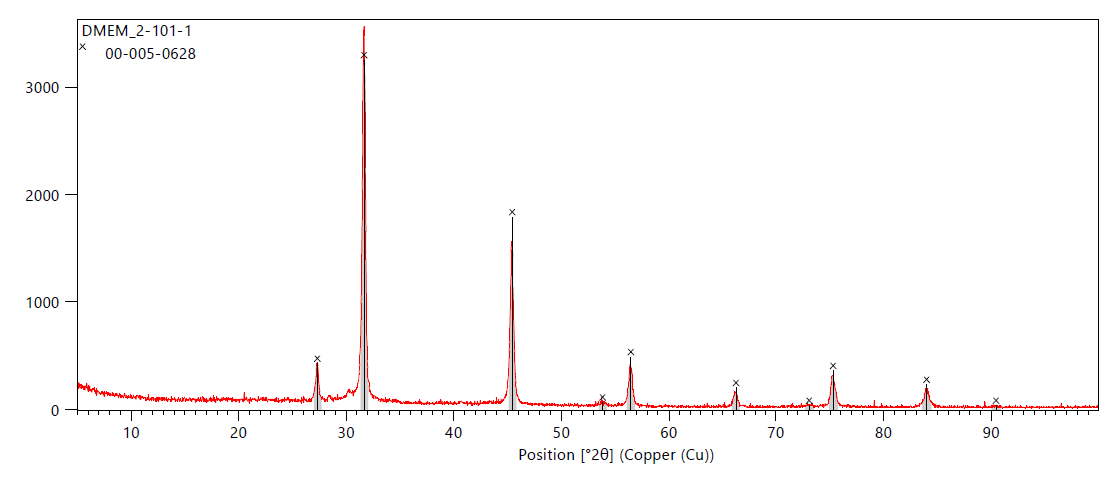


**Figure S8.** X-ray grazing incidence diffraction (GID) pattern of deposited DMEM aerosol particles, along with annotated NaCl reflections.

The selected area electron diffraction (SAED) pattern of a DMEM particle was collected using a JEOL 2100 TEM at 200 kV and a camera distance of 30 cm. A single crystal spot pattern of NaCl on [010] was superimposed with several rings with the same d-spacing as the spots, indicating that polycrystalline NaCl was also present. A weak additional single crystal is superimposed but was not strong enough for analysis. The amorphous region in the bright-field image (Figure S8a) is emphasized by the dark-field images (Figure S8c,d). This suggests that the main volume of the DMEM particles is a single region of NaCl crystal which also contains small polycrystals on the surface. The organic components (amino acids and dextrose) likely compose a concentrated amorphous region in the particle (bottom right) and also coat the surface to some extent. There are also notable crystals coating the surface which may be a combination of some NaCl crystals and also calcium crystals (from EDS mapped images). This was similar to what was observed in the cluster-analysed EDS maps (Figure S7).


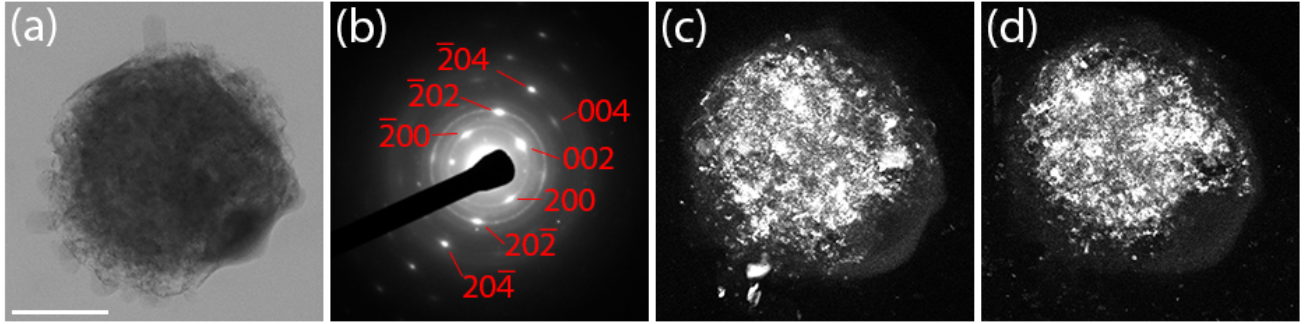


**Figure S9.** Selected area electron diffraction (SAED) of a dried DMEM aerosol particle. This figure includes (a) bright-field reference micrograph, (b) SAED pattern of the NaCl crystal on [010], (c) dark-field (DF) image of (002), and (d) DF image of ($\bar{\text{2}}\text{02}$).

## **S7. Infrared spectroscopy**

Dried DMEM particles were electrostatically deposited onto a Si wafer for several hours to produce a film. The infrared absorption spectrum of this film was measured using a Nicolet iS50 FTIR Spectrometer (ThermoFisher Scientific) in infrared reflection-absorption spectroscopy (IRRAS) configuration.


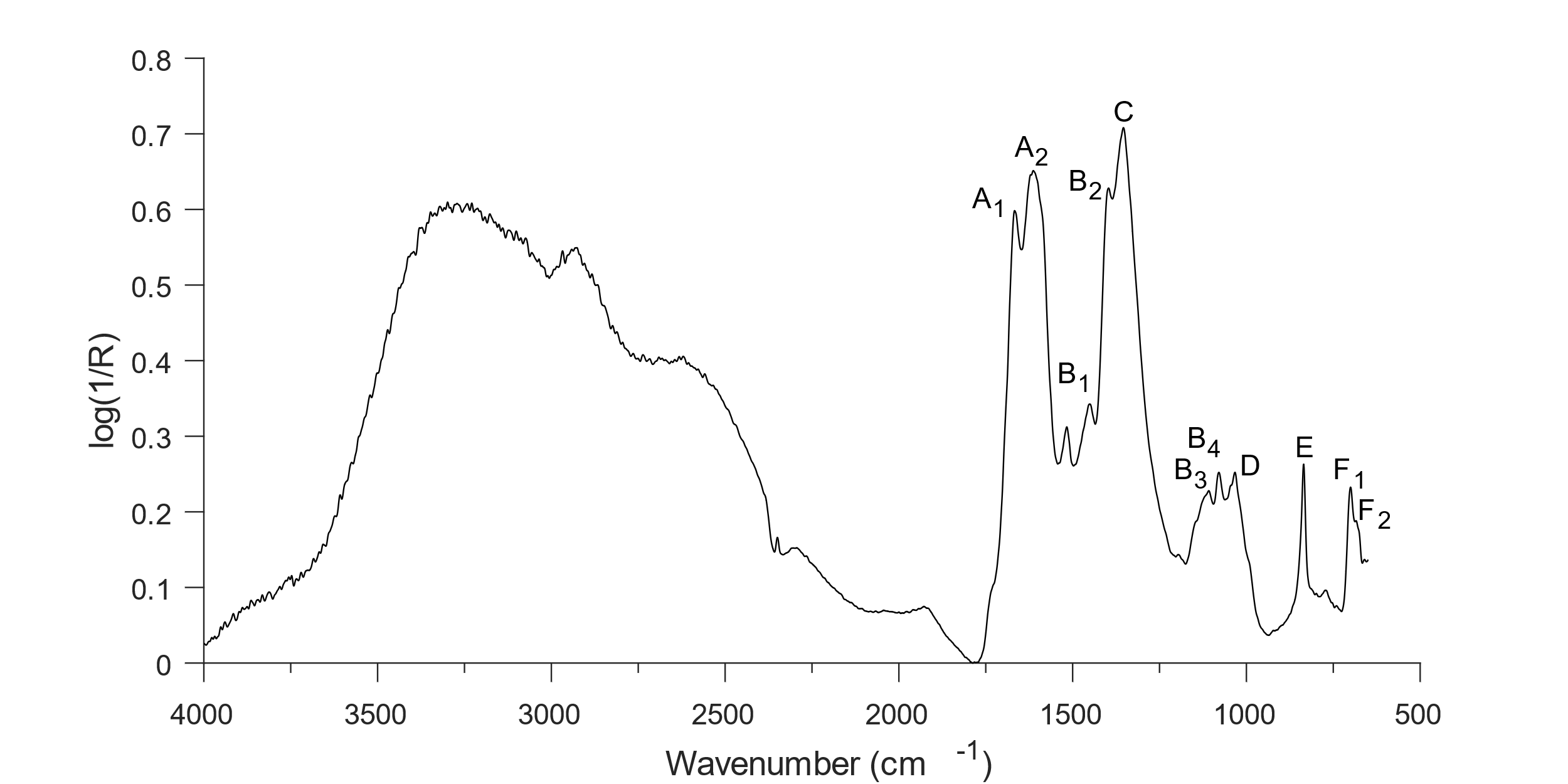


**Figure S10.** Infrared absorbance spectrum of deposited DMEM aerosol particles.

A sharp peak was measured at ~831 cm^-1^ (Figure S9, E) which is characteristic of $\text{H}\text{C}\text{O}_{\text{3}}^{\text{-}}$ out-of-plane bending. Also annotated in Figure S9 are the concomitant vibrational modes of NaHCO_3_ (C=O stretch → A_1_ and A_2_, mixed C-O and C=O stretches → B_1_ – B_4_, O-H-O in-plane bend → C, O-H-O out-of-plane bend → D, $\text{C}\text{O}_{\text{3}}^{\text{2-}}$ out-of-plane bend → E, mixed C=O and O-C-O bends & C-O and O-H stretches → F_1_ and F_2_). The peak positions of NaHCO_3_ were all blueshifted, suggesting isomorphic substitution of Na within the structure. The $\text{C}\text{O}_{\text{3}}^{\text{2-}}$ anti-symmetric stretching and out-of-plane bending doublets (~880 cm^-1^ and ~1420 cm^-1^, respectively) were not evident, indicating no $\text{C}\text{O}_{\text{3}}^{\text{2-}}$ in the sample. The broad symmetric bend (~1640 cm^-1^) of free water was not present, indicating that any residual water was limited to hydration water. Combined with the X-ray diffractogram (Figure S7), it is likely that any carbonate or acid carbonate species present were amorphous and had irregular ismorphically substituted cations as to not express resolved diffraction peaks.

## **References**

1. A. Zuend, C. Marcolli, B. P. Luo, T. Peter, A thermodynamic model of mixed organic-inorganic aerosols to predict activity coefﬁcients. *Atmos Chem. Phys.*, **35** (2008).

2. A. Zuend, *et al.*, New and extended parameterization of the thermodynamic model AIOMFAC: calculation of activity coefficients for organic-inorganic mixtures containing carboxyl, hydroxyl, carbonyl, ether, ester, alkenyl, alkyl, and aromatic functional groups. *Atmospheric Chem. Phys.* **11**, 9155–9206 (2011).

3. E. P. Vejerano, L. C. Marr, Physico-chemical characteristics of evaporating respiratory fluid droplets. *J. R. Soc. Interface* **15**, 20170939 (2018).

4. R. Groth, S. Niazi, G. R. Johnson, Z. Ristovski, Nanomechanics and Morphology of Simulated Respiratory Particles. *Environ. Sci. Technol.* **56**, 10879–10890 (2022).

5. R. Groth, L. T. Cravigan, S. Niazi, Z. Ristovski, G. R. Johnson, *In situ* measurements of human cough aerosol hygroscopicity. *J. R. Soc. Interface* **18**, 20210209 (2021).

6. E. Mikhailov, S. Vlasenko, R. Niessner, U. Poschl, Interaction of aerosol particles composed of protein and salts with water vapor: hygroscopic growth and microstructural rearrangement. *Atmospheric Chem. Phys.*, **28** (2004).

7. M. L. Pöhlker, *et al.*, Respiratory aerosols and droplets in the transmission of infectious diseases (2021) (June 8, 2022).

8. B. Münch, L. H. J. Martin, A. Leeman, Segmentation of elemental EDS maps by means of multiple clustering combined with phase identification. *J. Microsc*. **260**, (2015)
